# Heterologous production of 3-hydroxypropionic acid in *Methylorubrum extorquens* by introducing the *mcr* gene via a multi-round chromosomal integration system based on *cre-lox71*/*lox66* and transposon

**DOI:** 10.1186/s12934-023-02275-z

**Published:** 2024-01-03

**Authors:** Liping Zhu, Yazhen Song, Shunan Ma, Song Yang

**Affiliations:** 1 Shandong Province Key Laboratory of Applied Mycology, Qingdao International Center on Microbes Utilizing Biogas, School of Life Sciences, Qingdao Agricultural University, Qingdao, Shandong Province People’s Republic of China; 2 Key Laboratory of Systems Bioengineering, Ministry of Education, Tianjin University, Tianjin, People’s Republic of China

**Keywords:** Chromosomal integration, Cre, 3-hydroxypropionic acid, *M. Extorquens* AM1, Transposon, *lox71/lox66*

## Abstract

**Background and aim:**

Reprogramming microorganisms to enhance the production of metabolites is a part of contemporary synthetic biology, which relies on the availability of genetic tools to successfully manipulate the bacteria. *Methylorubrum extorquens* AM1 is a platform microorganism used to convert C1 compounds into various value-added products. However, the repertoire of available plasmids to conveniently and quickly fine-tune the expression of multiple genes in this strain is extremely limited compared with other model microorganisms such as *Escherichia coli*. Thus, this study aimed to integrate existing technologies, such as transposon-mediated chromosomal integration and *cre-lox-*mediated recombination, to achieve the diversified expression of target genes through multiple chromosomal insertions in *M. extorquens* AM1.

**Results:**

A single plasmid toolkit, pSL-TP-cre-km, containing a *miniHimar1* transposon and an inducible *cre*-*lox71*/*lox66* system, was constructed and characterized for its multiple chromosomal integration capacity. A co-transcribed *mcr-egfp* cassette [for the production of 3-hydroxypropionic acid (3-HP) and a reporting green fluorescent protein] was added to construct pTP-cre-mcr-egfp for evaluating its utility in mediating the expression of heterologous genes, resulting in the production of 3-HP with a titer of 34.7–55.2 mg/L by two chromosomal integration copies. Furthermore, in association with the expression of plasmid-based *mcr*, 3-HP production increased to 65.5–92.4 mg/L.

**Conclusions:**

This study used a multi-round chromosomal integration system based on *cre*-*lox71/lox66* and a transposon to construct a single constructed vector. A heterologous *mcr* gene was introduced through this vector, and high expression of 3-hydroxypropionic acid was achieved in *M. extorquens*. This study provided an efficient genetic tool for manipulating *M. extorquens*, which not only help increase the expression of heterologous genes in *M. extorquens* but also provide a reference for strains lacking genetic manipulation vectors.

**Supplementary Information:**

The online version contains supplementary material available at 10.1186/s12934-023-02275-z.

## Background

The methylotrophic bacterium *Methylorubrum extorquens* AM1 is an important platform organism for converting C1 compounds into various value-added products [1–6]. Therefore, achieving a high production or expression of heterologous genes, which, to a large extent, depends on the availability and diversity of genetic manipulation, is particularly critical. In the last two decades, several basic genetic strategies have been devised to facilitate the engineering of *M. extorquens* AM1, including one plasmid replicon system derived from broad-host-range IncP for heterologous or endogenous genes expression [7]; a broad-host-range suicide vector pCM433 for marker-free allelic exchange [8]; a *cre-lox* recombination system for antibiotic marker recycling [9]; several inducible promoters systems [10–13]; and mini-Tn5 derived transposon mutagenesis to identify genes involved in specific biosynthesis pathways [14–17]. However, co-expressing multiple genes in this strain only depends on the solitary IncP-based vector at very low copy number expression [11]. This is far inferior to the multiple compatible replicon types present in *Escherichia coli*, which functionally distributes heterologous genes to different plasmids for co-expression [18, 19]. Thus, the strategy to facilitate the co-existence of multiple expression plasmids can improve the operability of *M. extorquens* AM1. Considering the difficulty of developing new genetic technologies, integrating existing technologies may provide a feasible solution.

When it comes to the expression of heterologous metabolic genes, plasmid-based systems and chromosomal integration are two distinct strategies commonly used [20]. The plasmid-based system used in industrial fermentation potentially leads to extra metabolic burden and genetic instability, and therefore, the chromosomal integration of exogenous genes is preferred for fermentation [20, 21]. Among the technologies mentioned earlier in *M. extorquens* AM1, transposons with a broad host feature can permanently mediate chromosomal integrations. This capability allows for creation of transposon knockout mutant libraries or stable insertion of a specific gene of interest at a specific chromosomal site. As a result, it becomes a valuable discipline for bacterial engineering studies [19, 22–25]. Three transposons have been used in *M. extorquens* AM1: mini-Tn5 and *miniHimar1*, as a random insertion transposon [14–17, 26]; and *mini-*Tn7, which evokes a site-specific and single-copy chromosomal insertion at the attTn7 site [27]. The Mu, Tn7, and Tn3 family transposons have been approved to have the target immunity effect, which means that the transposon inserted into the genome only once thereby preventing repeated insertion of the same transposon [28, 29]. However, no study explored the target immunity effect of random transposons. Moreover, the position effect of different insertion sites on the genome caused by the random insertion transposon results in heterologous production diversity [21, 22, 30, 31]. Therefore, specific strains capable of producing particular amounts can be selected from the transposon-based transformants as required.

In this study, we aimed to perform multi-round integrations of target genes using *miniHimar1*, a random insertion transposon that only requires a TA dinucleotide sequence for transposition [26, 32]. Simultaneously, we facilitated multiple chromosomal insertions via a single plasmid using a *cre-loxP* recombination system [9, 33], which is essential for the scarless elimination of the antibiotic marker. The *cre-loxP* recombination system was previously established in *M. extorquens* AM1, but in two separate vectors [9]. Another application in *M. extorquens* AM1 was the integration of tandem multicopy of the mevalonate operon into the genome [29]. However, constant expression of Cre protein may lead to excessive integration of *loxP* sites into the genome, thereby reducing the copy number. Therefore, an inducible promoter for *cre* is needed, as previously described [9]. In addition, *lox66* and *lox71* sites were recruited instead of *loxP*, forming a hybrid lox71/66 site after recombination. This could prevent repeated recombination between different *lox* sites and instability caused by multiple *loxP* sites in the genome [34, 35].

This study was novel in reporting a multiple chromosomal integration system based on *cre-lox71/lox66* and transposon. The co-transcribed *mcr* and *egfp* were proposed, which encoded a malonyl-CoA reductase for converting methanol into 3-HP and a reporter green fluorescent protein for high-throughput screening, respectively. These genes were inserted into the chromosome of *M. extorquens* AM1 with expected copy numbers. Finally, a high 3-HP production was achieved in *M. extorquens* AM1 by combining the transposon-mediated chromosomal integration and plasmid-based systems.

## Results

### Construction of integration vectors

**Fig. 1:**
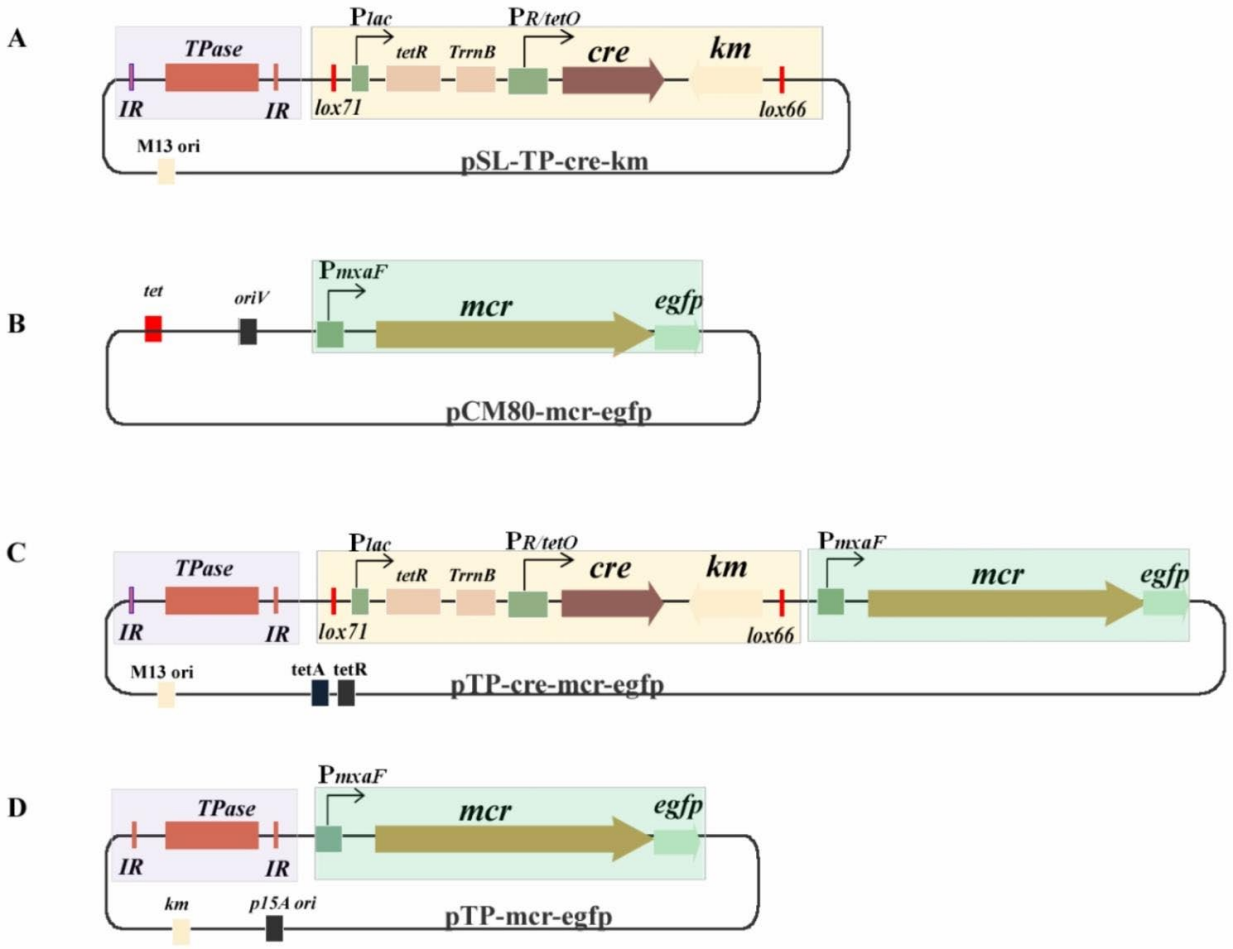
Schematic diagram of vectors constructed in this study. **(a)** pSL-TP-cre-km; **(b)** pCM80-mcr-egfp; **(c)** pTP-cre-mcr-egfp; **(d)** pTP-mcr-egfp *Tpase*: *miniHimar* transposase. *IR*: insertion sequence recognized by *miniHimar* transposase. *TrrnB*: transcriptional terminator. *PR/tetO*: inducible promoter by aTc. *cre*: gene coding for Cre recombinase. *km*^*r*^: kanamycin resistance gene. *egfp*: gene coding for green fluorescent protein. *tet*: tetracycline resistance gene. M13 *ori*: high-copy replicon origin for *E. coli*. *p15A ori*: low-copy replicon origin. *lox71*and *lox66*: the lox sites for recombination by Cre

In this study, a chromosomal integration system based on the inducible *cre-lox71/lox66* recombination system and *miniHimar1* transposon was constructed in order to explore the potential for multiple integrations of heterologous genes in the chromosome of *M. extorquens* AM1. All the physical maps of the integration vectors constructed in this study are shown in Fig. 1 and Fig. S1, and the construction processes are illuminated in Fig. S2–S4. First, pSL-TP-cre-km, which contained the fundamental elements of transposon and recombinase, was constructed to be a versatile tool. Briefly, *miniHimar*1, as a mariner-origin transposon, was recruited for an unbiased random insertion of heterologous genes into the genome of *M. extorquens* AM1. The Cre recombinase, followed by a kanamycin selectable gene (km), was regulated by an inducible hybrid promoter *PR/tetO* [12], which initiated gene recombination at *lox71* and *lox66* sites when triggered by aTc, leading to the excision of *cre* and km from the genome. In this case, the same vector or another plasmid with a km resistance gene could potentially enter the cell again. Based on the *miniHimar1* transposon and *cre-lox71/66* recombination system, we expected to achieve multiple integrations of heterologous genes into the genome of *M. extorquens* AM1. Finally, the *mcr* gene responsible for the production of 3-hydroxypropionic acid was recruited to construct pTP-cre-mcr-egfp in this study, and a reporter *egfp* gene co-transcripted with *mcr* was used for a high-throughput screening of the target strains. As a control, pCM80-mcr-egfp, generated by cloning *mcr* and *egfp* into pCM80 and expressed in a multicopy extranuclear plasmid form, was constructed. Meanwhile, pTP-mcr-egfp without cre was also constructed for one entrance of *mcr-egfp*.

**Fig. 2:**
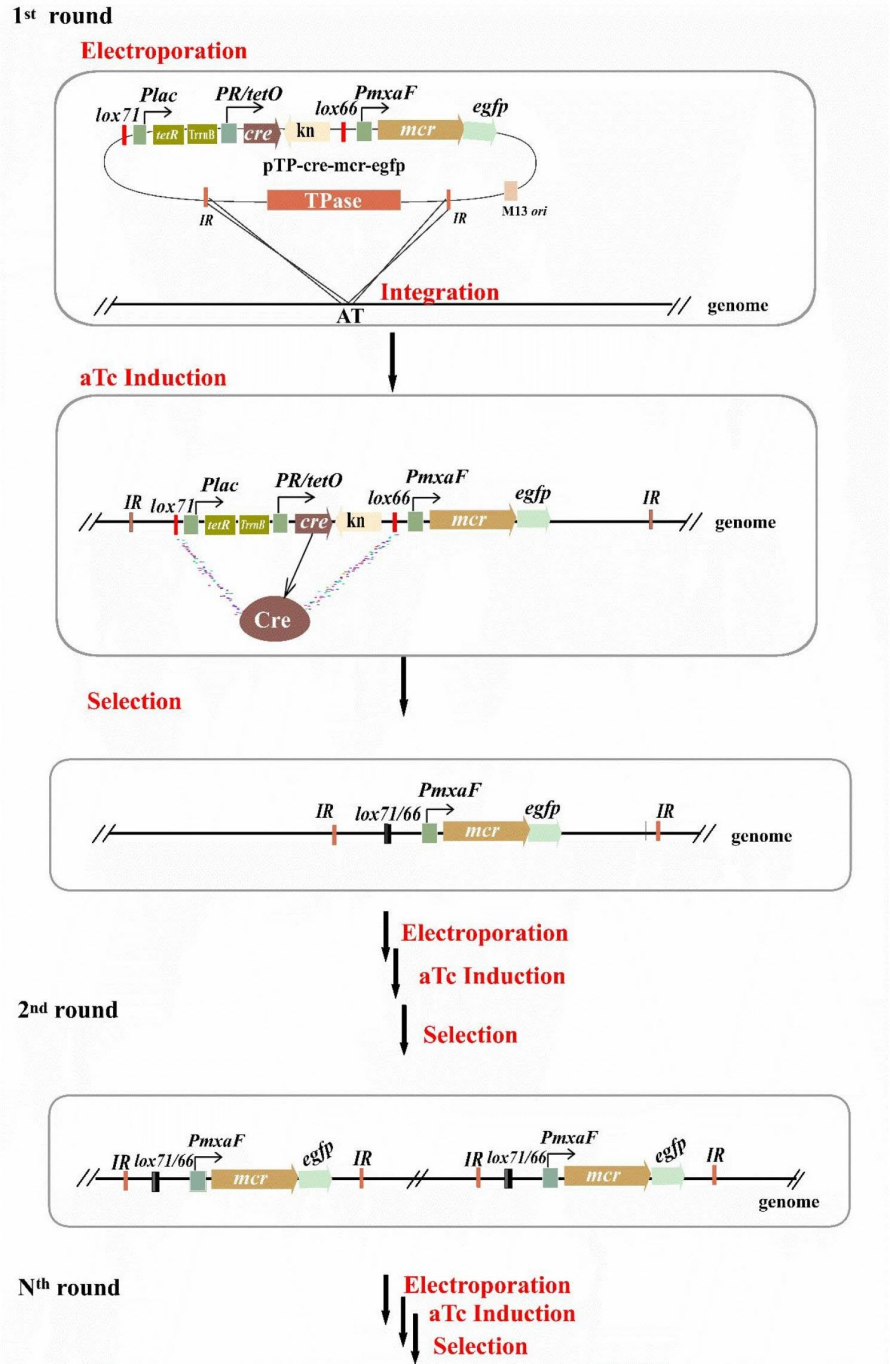
Mechanism of multiple genome integration system based on transposon-mediated *cre*-*lox71/66* recombination

The mechanism of this multiple chromosomal integration system is illustrated in Fig. 2. In the first round, a target plasmid such as pTP-cre-mcr-egfp was electroporated into *M. extorquens* AM1 competent cells, and genes between the two *IRs* were integrated into the genome via transposon. After adding anhydrotetracycline (aTc), the Cre bound to the *lox71* and *lox66* sites, and allow recombination between these two lox sites. This meant that the *cre* and *km* were eliminated from the chromosome, leaving a hybrid lox71/66 site that was no longer recognized by Cre. Simultaneously, the target *mcr* along with *egfp* was left in the chromosome. As the random integration via transposon could result in a differential expression of heterologous genes [22, 30, 36], strains with the correct phenotype and higher levels of 3-HP production or fluorescence intensity were selected and used as hosts for the second round, and so on. This approach allowed us to obtain a strain with multiple copies of the target *mcr* gene, as expected.

### Verification of the feasibility of different replicons and transposon immunity of miniHimar1 in *M. extorquens* AM1

The *p15A* replicon has been proved to be deficient in *M. extorquens* AM1 in a previous study [26]. Therefore, plasmid p15A-M6.8 can only integrate into the genome via *miniHimar1* transposon. The replicon of pSL1180 is an *E. coli–*derived M13 *ori*. Also, pSL-km was constructed to test its feasibility in *M. extorquens* AM1. Without exception, we did not obtain any colonies on the screen plate after the electroporation of pSL-km. This indicated that the M13 *ori* is also deficient in *M. extorquens* AM1. Therefore, the transformant colonies that survived on kanamycin plates after the electroporation of pSL-TP-cre-km and pTP-cre-mcr-egfp into *M. extorquens* AM1 were resorted to transposon integration into the genome by *miniHimar1*. The IncP*-*derived *oriV* replication origin of pCM80-mcr-egfp allowed the expression of *mcr* as an extranuclear plasmid with a low copy number.

Although *miniHimar1* has been used in numerous organisms [37], but no effect of transposon immunity has been reported. A former *miniHimar1* containing mutant *M. extorquens* AM1(*lacZ*), which was constructed by transforming p15A-M6.8 into *M. extorquens* AM1 [26], was used as a host to transform the pTP-cre-mcr-egfp carrying the same *miniHimar1*, and kanamycin and another tetracycline resistance gene, so as to verify the transposon immunity. Abundant colonies were obtained in the dual resistance plate, indicating no transposon immunity in this *miniHimar1*. Therefore, *miniHimar1* transposon can be repeatedly employed for chromosomal integration.

### Function analysis of the *cre*-*lox66/lox71* system

As previously reported, Cre associated with two *loxPs* expressed in the plasmid has been used in *M. extorquens* AM1 [3, 9]. This study, first used *lox66* and *lox71* sites to avoid interactions between multiple *loxPs*. To verify the functionality of the *cre*-*lox71/lox66* system, we tested the pSL-TP-cre-km which carries the inducible *cre* and *lox71/lox66* sites in *E. coli* at the plasmid level. Since plasmid extraction from *M. extorquens* AM1 remains unfeasible, a smaller plasmid was obtained after inducing Cre, resulting in the excision of *cre* and *km* by recombination (Fig. S5a). Then, pSL-TP-cre-km was transformed into *M. extorquens* AM1 and the transformants named *M. extorquens* AM1-TC were used for the inducible expression of Cre. Finally, colonies that lost their growth ability in the presence of kanamycin were obtained and determined to be *M. extorquens* AM1-T, indicating that the *cre*-*lox71/lox66* was effective in *M. extorquens* AM1 (Fig. S5b). The inducible recombination efficiency was calculated to be 73.4%, as shown in Fig. S5b. It was confirmed by colony-polymerase chain reaction (PCR) that the gene elements between *lox71* and *lox66* sites (including *cre* itself and *km*) were excised from the genome (Fig. S5c). The colonies exhibited no discrepancy in growth with the wild host *M. extorquens* AM1 (Fig. S5d).

### Co-transcribed *mcr-egfp* cassette

**Fig. 3:**
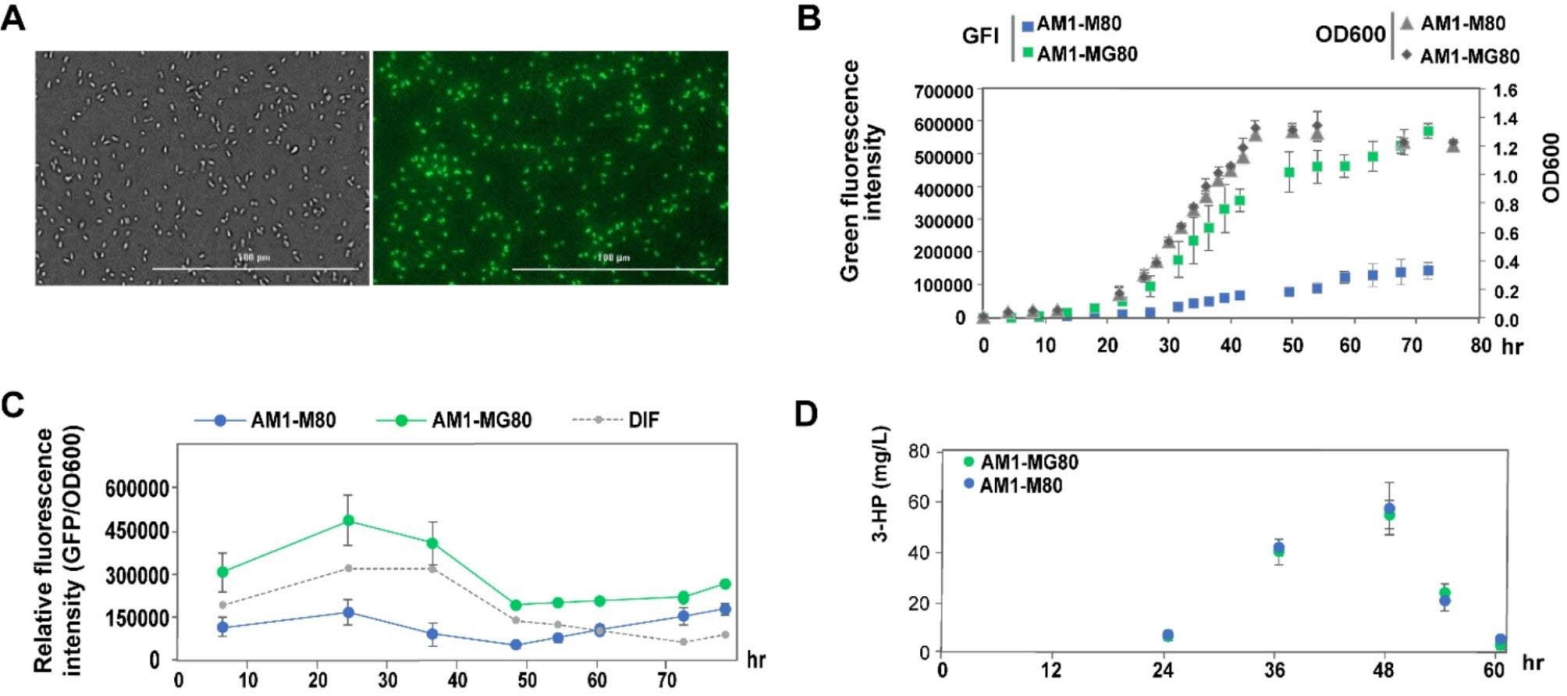
Fluorescence assay and 3-HP detection of the transformant AM1-MG80. Strain AM1-M80 was used as control. **(a)** Fluorescence microscopic observation of AM1-MG80 cells at bright field (left) and green fluorescent channel (right). **(b)** Growth curve and total green fluorescence intensity of AM1-MG80 and AM1-M80. The horizontal axis hr represents the time of hour, and the vertical axis represents the value of fluorescence intensity (left) and the value of OD_600_ (right). **(c)** Relative fluorescence intensity (RFI) of AM1-MG80 and AM1-M80. DIF indicates the difference in RFI between AM1-MG80 and AM1-M80. The vertical axis represents the fluorescence intensity per OD. **(d)** Variation in 3-HP production of AM1-MG80 and AM1-M80 with cell growth

The co-transcribed *mcr-egfp* cassette was constructed in pCM80-mcr-egfp by fusing a promoter-less *egfp* downstream of the *mcr* gene to establish a fluorescence reporter system for 3-HP. pCM80-mcr without *egfp* was used as a control. The transformants of pCM80-mcr-egfp and pCM80-mcr, referred to as AM1-MG80 and AM1-M80, respectively, were selected to measure fluorescence and 3-HP levels (Fig. 3). The AM1-MG80 was detected with green fluorescence, as illustrated in Fig. 3a. The total fluorescence intensity increased along with cell growth (Fig. 3b), whereas the relative fluorescence intensity (RFI) showed a peak in the exponential phase (Fig. 3c). The 3-HP levels in AM1-MG80 and AM1-M80 were detected by high-performance liquid chromatography (HPLC) with an expected peak production of ≈ 60 mg/L (Fig. 3d and S6). It is worth noticing that 3-HP has been previously identified with a rapid degradation at the transition from the exponential phase to stationary phase [4], indicating that 3-HP production cannot continue to increase with the cell growth (Fig. 3d). Therefore, the relationship between 3-HP production and fluorescence expression intensity should be further investigated in this regard.

### Analysis of position effect in the transposon-mediated single chromosomal integration recombinants

**Fig. 4:**
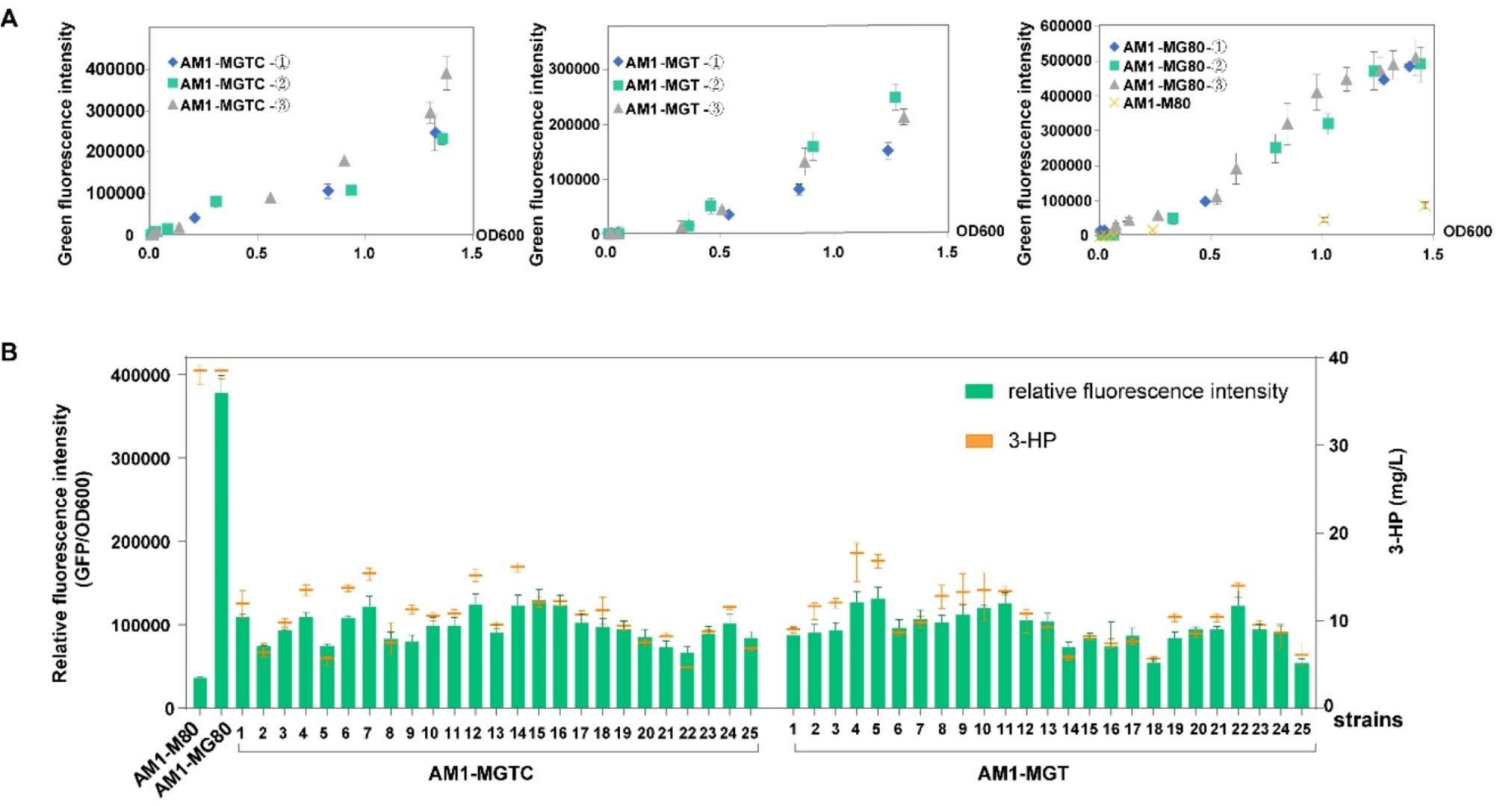
Fluorescence assay and 3-HP detection of the isolates of AM1-MGTC and AM1-MGT. **(a)** Fluorescence change with OD_600_ before the stationary stage in one of the isolates of AM1-MGTC and AM1-MGT, each with triple repeats. **(b)** Fluorescence and 3-HP production of the isolates of AM1-MGTC and AM1-MGT; those of AM1-MG80 and AM1-M80 were used as controls. The columns indicate RFI and the dots indicate the 3-HP production. The numbers 1 to 25 stand for different isolates of recombinant AM1-MGTC and AM1-MGT.

Considering the difference in heterologous gene expression caused by transposon insertion site effects [22, 30, 31], the *mcr-egfp* cassette was used as an indication system for the transposon-mediated position effect. For this, pTP-cre-mcr-egfp and pTP-mcr-egfp, containing both the transposon and the *mcr-egfp* cassette, were transformed into *M. extorquens* AM1, and the corresponding single chromosomal integration recombinants were named AM1-MGTC and AM1-MGT, respectively. Before the follow-up experiments, the sampling time points were required to be determined, so that three isolates of each transformant could be used for detecting fluorescence intensity and growth OD_600_ till the stationary stage. The result showed that the change in the fluorescence of transformants was associated not only with cell OD_600_ but also with an obvious fluctuation between the parallels since OD_600_ was ≈ 0.8 (Fig. 4a). Nevertheless, the plasmid-based AM1-MG80 showed a stable change between the parallels (Fig. 4a). OD_600_ ≈ 0.8-1.0, indicating the late exponential phage, was preferred for most enzymatic activity or metabolomic analysis in *M. extorquens* AM1 transformants [26, 38]. Taken together, the fluorescence detection in the late exponential phase with an OD_600_ of 0.9 ± 0.1was used to determine whether it was competent for a high-throughput reporting system for 3-HP production. Subsequently, 25 isolates from each transformant were selected for detecting the fluorescence intensity and 3-HP production. As expected, different isolates displayed fluctuating expression levels, as shown in Fig. 4b, which was mainly attributed to the influence of genome insertion sites through the transposon instead of the biological differences [22]. Nonetheless, we observed a positive correlation between the expression of fluorescence intensity and 3-HP production within the corresponding isolates. This suggested that co-transcribed *mcr* and *egfp* regulated transcriptional levels in the cassette in a synchronized manner. In addition, the transposon recombinants showed a low expression of *mcr-egfp* genes when compared with AM1-MG80, which had multiple copies via an extranuclear plasmid as a control, due to the single insertion copy by the transposon (Fig. 4b). Thus, an effective fluorescence reporter system for the high-throughput screening of heterologous gene expression, such as *mcr*, was developed in this study. The isolates with a high 3-HP production were further executed for the second round of transformation.

### Induction and second-round chromosomal integration

The no. 14 of the recombinant AM1-MGTC, which exhibited a relatively high expression of *mcr-egfp* (25.7 mg/L of 3-HP, which was 0.42 times that of strain AM1-MG80), was selected as the host for the second round of genome integration. First, the *cre* and *km* elements within AM1-MGTC were eliminated from the genome after induction by aTc. The corresponding colonies were identified and verified on plates with and without kanamycin. The no. 14 of the recombinant AM1-MGTC displayed an inducible recombination efficiency of 27.1%, which was far less than that of the recombinant with pSL-TP-cre-km mentioned above (Fig. S7a). This was mainly attributed to the existence of an extra *tetR* gene, which was located in front of *tetA* introduced by pTP-cre-mcr-egfp in AM1-MGTC. The TetR repressor encoded by *tetR* could interact with the promoter of *tetA* or *tetO* (Fig. S7b) and repress its expression. Meanwhile, the TetR repressor had a strong affinity for tetracycline, inactivated tetracycline and anhydrotetracycline (aTc, a high-affinity ligand) at extremely low concentrations [26, 39, 40]. The inducible *PR/tetO* promoter was dose-dependent with a 30-fold regulatory range when responding to a range of 0.1– 50 ng/mL of aTc [12]. Therefore, we increased the concentration of aTc from 25 ng/mL to 50 ng/mL, and then the recombination efficiency up increased to 64.2% (Fig. S7a), suggesting that we should either increase the concentration of inducer or switch to another antibiotic resistance marker instead of *tetA*.

**Fig. 5:**
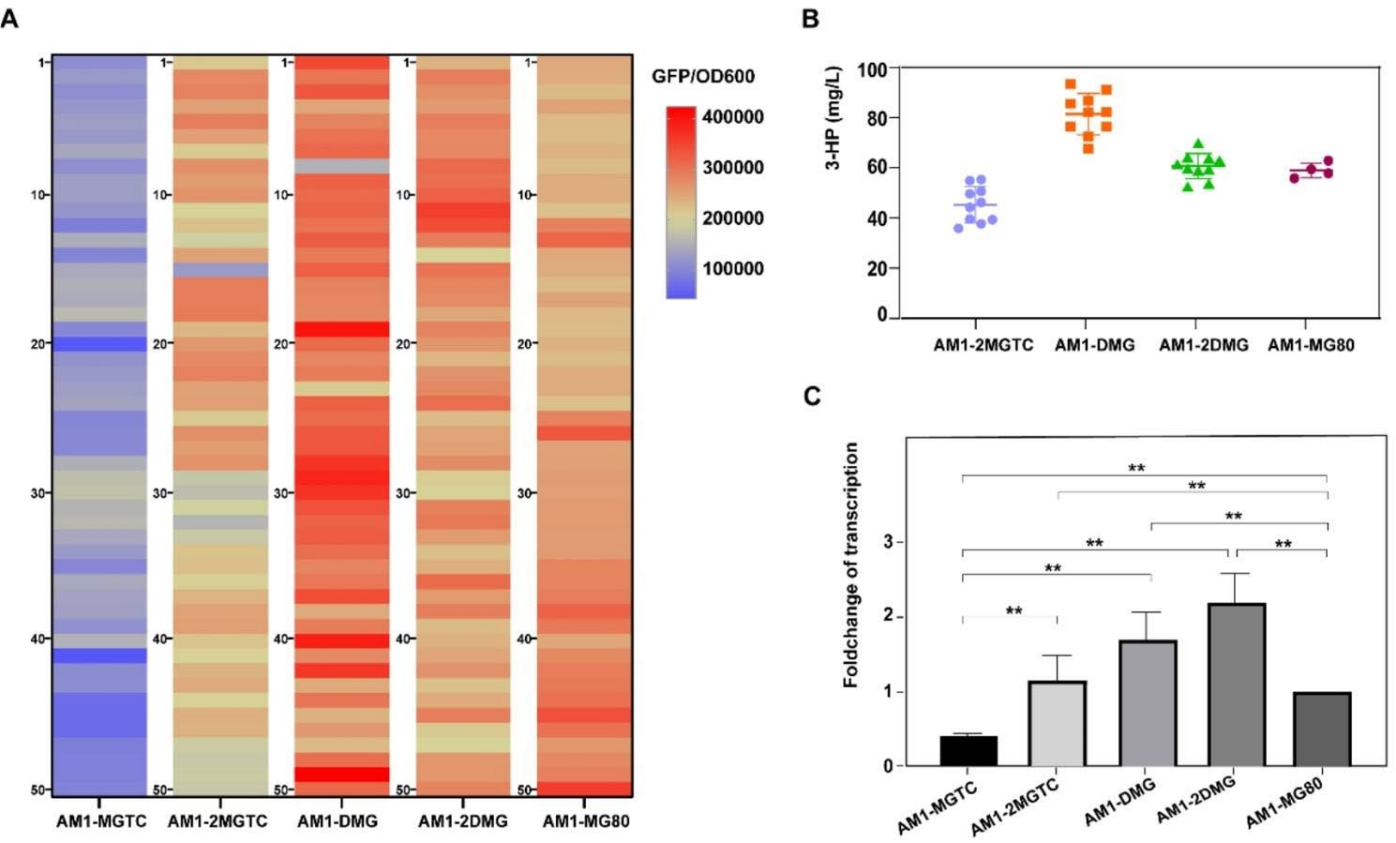
Comparative analysis of fluorescence and 3-HP detection of all the constructed strain isolates. **(a)** Heatmap of RFI generated from 50 picked isolates of all constructed strains. The numbers on the left of each heatmap column represent the strain isolates, and the color column on the upper right indicates the RFI values (GFP/OD_600_). **(b)** 3-HP production of the top 10 isolates of each strain based on the RFI, and strain AM1-MG80 (4 isolates) was used as control. **(c)** qRT-PCR analysis of transcriptional expression of the *mcr* gene in all the constructed strains. The expression level of genes in AM1-MG80 strains was set as 1. the *t*-test was used to calculate the statistical significance by pairwise comparison. * 0.01 < *P* < 0.05; **P < 0.01

After induction, the *cre*-*km* eliminated mutants screened from the photocopying plate were confirmed by colony PCR, fluorescence intensity, and 3-HP production detection. The corrected colonies were then used as hosts for the second round of transformation of pTP-cre-mcr-egfp to make transformant AM1-2MGTC. Further, 50 colonies of AM1-2MGTC were picked, and their fluorescence intensities were significantly increased compared with those of AM1-MGTC colonies (Fig. 5a). The top 10 strains were chosen for 3-HP detection, and all of them produced 3-HP at levels from 34.7 mg/L to 55.2 mg/L (Fig. 5b).

### Combination use of two types of the expression plasmid

Next, we attempted to combine the transposon integration plasmid and the extranuclear plasmid. On the one hand, pTP-cre-mcr-egfp was transformed into the host AM1-MG80 to make the recombinant strain AM1-DMG. Then 50 colonies were picked, which showed the highest fluorescence intensity among all the constructed strains. The top 10 isolates exhibited a 3-HP production capacity ranging from 65.5 mg/L to 92.4 mg/L, representing the highest yield in this study. However, when plasmid pCM80-mcr-egfp was transformed into the candidate isolate of AM1-2MGTC, no more significant increase in fluorescence intensity or 3-HP production was observed in the corresponding colonies (AM1-2DMG) compared with those of AM1-MG80 (Fig. 5a and b). This might be due to the metabolic restriction of *M. extorquens* AM1 or the transcriptional obstacle of the *mcr* gene. Therefore, quantitative Real-Time PCR (qRT-PCR) was performed to demonstrate that the transcriptional level of *mcr* was positively correlated with gene copy numbers but was not exactly consistent with the 3-HP production in each constructed strain type, especially in the combination expression strains (Fig. 5b and c). Specifically, the strain AM1-2DMG, which simultaneously contained two copies of chromosome-integrated *mcr* and an extracellular plasmid-based *mcr*, showed the highest transcriptional expression, but a lower yield of 3-HP compared with AM1-DMG, which had one less integrated *mcr* copy number. However, AM1-2MGTC displayed 2.9-fold more expression compared with AM1-MGTC, beyond the ratio of integrated gene copy number. Again, these results demonstrated that not only gene copy numbers, but also chromosomal integration sites had an important influence on gene transcriptional expression levels.

## Discussion

Several genetic operation elements have been developed for *M. extorquens* AM1, a model methylotrophic strain with potential for industrial application [7, 9, 12, 41]. However, using only IncP-derived extranuclear plasmids and the insufficiency of antibiotic-resistance markers have limited the genetic manipulation and application of *M. extorquens* AM1 in industrial biotechnology. Moreover, plasmid-based systems typically lead to high- and low-performance variants in culture due to cell-to-cell variations caused by plasmid genetic instability, which affects their suitability in large-scale or long-term industrial fermentation [20, 42]. Therefore, chromosomal integration is a preferable approach, as genes can be stably maintained without selective antibiotics and exhibit consistent gene expression at a single-cell level [43]. For prokaryotic microbes, the genomic integration of foreign genes is usually mediated by homologous recombination (HR) [44] and site-specific recombination (SSR) [45]. Compared with HR, SSR is much more time- and effort-saving, but it requires a selection marker to identify its occurrence. Thereafter, the targeted integration site is destroyed, hindering the multi-round integration of foreign genes. However, transposon-mediated chromosomal integration involves multi-round integration because of no perference for insertion sequence. Furthermore, the transformants generated after transposition result in different expression levels of foreign genes due to the position effect caused by adjacent genes [22, 30, 31], providing a new idea for screening strains with a certain level of expression or yield as required. Among all the transposons used in *M. extorquens* AM1, the *miniHimar*, derived from an extensively used mariner transposon with a broad host range [22, 46], was identified with no transposon immunity in *M. extorquens* AM1. It was then used to construct a multi-round chromosomal integration system in this study. In particular, the *cre*-*loxP* system, a well-known recombination method for eliminating the resistance marker without a scar [19, 47–49], was recruited to resolve the selection marker issue caused by synchronous integration. With modification, the inducible *cre-lox71*/*lox66-km* cassette was constructed, facilitating a stable integrated copy number, allowing for the reuse of the selective marker (*km* resistance gene), and also protecting the host from the toxic effects of continuous Cre expression. With these initiatives, two rounds of integration were achieved through a single constructed vector (pSL-TP-cre-km), thus expanding the genetic manipulation capacity of *M. extorquens* AM1.

A heterologous *mcr* gene was introduced to produce 3-HP via the malonyl-CoA pathway to test and verify the application of the constructed pSL-TP-cre-km system. Before that, the established co-transcribed *mcr*-*egfp* demonstrated a positive correlation between 3-HP production and fluorescence intensity at specific optical density points; *egfp* was normally used as a high-throughput screening indicator. Accordingly, *mcr*-*egfp* was used to construct an expression vector in the form of pTP-cre-mcr-egfp. After the first round of transformation, isolates (AM1-MGTC) were identified with 3-HP titers ranging from 0.12 to 0.46 times that of strain AM1-MG80, which was designated as the original plasmid-based expression. Among these, the isolate with the highest titer was consistent with the measured *mcr* transcription level and thus served as a host for the second round of transformation after induction (Figs. 4 and 5). The maximum 3-HP production from the transformants of the second integration (AM1-2MGTC) increased to 55.2 mg/L, which was 90% of the original plasmid-based yield. These results suggested that the specific titer was enhanced when an extra copy of *mcr* was integrated into the chromosome. However, the most productive isolate of AM1-2MGTC was proportional to the gene copy number, when compared with its host. Nonetheless, it was quite close to the production of plasmid-based AM1-MG80, which contained the plasmid with eight copy numbers in general [11]. It was speculated that the integration site effect overrode the influence of plasmid-based gene copy numbers and the plasmid-based overexpression was already a burden to *M. extorquens* AM1, leading to a slight increase in 3-HP production in the combination expression strains (AM1-DMG and AM1-2DMG). So far, the highest 3-HP production reported in *M. extorquens* AM1 was 91.2 mg/L, which was based on a constructed ribulose monophosphate pathway cycle and intrinsic serine cycle [38]. A similar titer of 92.4 mg/L was obtained with the AM1-DMG strain constructed by the combination expression strategy. The comparison of 3-HP titer and gene transcriptional activity between AM1-DMG and AM1-2DMG revealed a deficiency, which was caused by excessive copies that overwhelmed the host cell and impaired the metabolic capacity or by the insufficiency of the rate-limiting enzymes for the pathway [50, 51]. The production of 3-HP requires the consumption of one molecule of acetyl-CoA, one molecule of ATP, and two molecules of NADPH [4]. More in-depth and detailed experiments need to be executed to verify the findings.

The *mcr* gene copy number in AM1-2MGTC was increased after two rounds of chromosomal integration, and the corresponding 3-HP titer was much higher than that in AM1-MGTC. This indicated that the chromosomal integration of multiple gene copies had a significant effect in terms of improving the gene copy number and its production. In other words, increasing the gene copy numbers through multiple rounds of integration was an effective approach for heterologous expression under the influence of integrated sites of transposon within a certain limit. A large number of studies proved that chromosomal multiple position integration strategy could be more efficient in heterologous biosynthesis, whether integrating tandem copies of the same gene into a specific site or integrating different genes into different sites [29, 52]. Also, it is possible to observe a bipolar effect of different integration sites, either positive or negative, on the titer based on the differences between individual isolates of certain transformants. Therefore, the insertion sites of the transformants need to be determined in follow-up experiments to provide more rational interpretation and guidance.

## Conclusion

This study constructed a multicopy chromosomal integration expression system in *M. extorquens* AM1 based on an inducible *cre-lox71/lox66* recombination system and *miniHimar1* transposon. The system allowed for rapid insertion of the heterologous *mcr* gene and the creation of mutants with high levels of corresponding 3-HP yield, with the resistance gene eliminated. This is beneficial for industrial applications. The extensive distribution of the random insertion sites also provided additional references for screening high-expression strains. Finally, we could elevate 3-HP titer in *M. extorquens* AM1 using a combination of one-round chromosomal integration and an extranuclear plasmid. Our findings will extend the genetic manipulation of *M. extorquens* AM1 and improve its potential for heterologous expression.

## Materials and methods

### Bacterial strains and culture conditions

*Escherichia coli* DH5α used for routine transformations and subcloning was grown at 37℃ in a Luria Broth medium supplemented with antibiotics [ampicillin (Amp), 100 µg/mL; kanamycin (Km), 40 µg/mL; tetracycline (Tet), 10 µg/mL], for selecting the constructed plasmids. The heterologous host used for fluorescent protein reporter genes *egfp* and *mcr* was *M. extorquens* AM1, which was grown at 30℃ in a medium, containing 0.5% (v/v) methanol (123 mM), as well as 3.31 g/L K_2_HPO_4_·3H_2_O, 1.68 g/L NaH_2_PO_4_·2H_2_O, 0.2 g/L MgSO_4_·7H_2_O, 0.5 g/L (NH_4_)_2_ SO_4_ and the following trace elements: 10 mg/L Na_2_EDTA, 1 mg/L FeSO_4_·7H_2_O, 1.4 mg/L CaCl_2_·2H_2_O, 1 mg/L MnCl_2_·4H_2_O, 0.2 mg/L Na_2_MoO_4_·2H_2_O, 0.3 mg/L CuSO_4_·5H_2_O, 1.6 mg/L CoCl_2_·6H_2_O, and 4.4 mg/L ZnSO_4_·7H_2_O, with or without the antibiotics before or after introduction of the relevant genes. pSL1180 and p15A-M6.8, proved to be suicide plasmids in *M. extorquens* in this study, were used for vector construction in *E. coli.* The IncP replicon-based plasmids pCM80, pCM157, pLC291, pCM80-mcr, and pCM80-egfp were used as the original vector for further construction. All vectors constructed in this study are listed in Table 1. Especially, pCM80 and pSL1180 were used as backbones for a series of vector construction; p15A-M6.8 provided the *p15A* replicon, *miniHimar1* transposon and *lacZ*; pCM157 provided the *cre* gene; pLC291 provided the promoter *PR/tetO* induced by aTc; and pX458 provided the fluorescent gene *egfp*.

**Table 1:** Plasmids and strains used in this study

Plasmids/Strains	Characteristics	Source or reference
***Plasmids***		
pSL1180	*ColE* ori; Amp^r^	Pharmacia
p15A-M6.8	*p15A* ori; *lacZ*; *miniHimar1* transposon elements; km^r^	[26]
pCM80	Universal plasmid used in *M. extorquens*, with a *PmxaF* promoter; Tet ^r^	[7]
pCM157	Contains the constitutively expressed *cre* gene; Tet^r^	[9]
pLC291	Contains *PR*/*tetO* inducible promoter; km^r^	
pCM80-mcr	*mcr* gene was connected next to the *PmxaF* promotor on pCM80; Tet ^r^	This study
pCM80-eGFP	*egfp* gene was connected next to the *PmxaF* promotor on pCM80; Tet ^r^	This study
pCM80-mcr-eGFP	*egfp* co-transcribed with *mcr* was linked to pCM80; Tet ^r^	This study
pLC-Cre	*cre* gene was connected next to *PR*/*tetO* inducible promoter on pLC292; km^r^	This study
pSL-TP	*miniHimar1* transposon elements was linked to pSL1180; Amp^r^	This study
pSL-TP-cre-km	*cre* and *lox71*/ *lox66* sites were linked to pSL-TP; Amp^r^; km^r^	This study
pTP-cre-mcr-egfp	Contains *p15A* ori; *miniHimar1* transposon elements; *PR*/*tetO* inducible promoter; *cre*- *lox71/lox66* cassette; *mcr*-*egfp*; km^r^	This study
pTP-mcr-egfp	Contains *p15A* ori; *miniHimar1* transposon elements; *mcr-egfp*; km^r^	This study
***Strains***		
*M. extorquens* AM1	Original host for the production of 3-HP	[7]
*M. extorquens* AM1(*lacZ*)	Transformant of *M. extorquens* AM1 with chromosomal insertion of *lacZ* gene by p15A-M6.8	[26]
*M. extorquens* AM1-TC	Plasmid pSL-TP-cre-km was transformed into *M. extorquens* AM1, km^r^	This study
*M. extorquens* AM1-T	*M. extorquens* AM1-TC was induced to perform recombination at the *lox71* and *lox66* sites, resulting in the removal of *cre-km* cassette	This study
AM1-MG80	Transformant of *M. extorquens* AM1 with plasmid-dependent *mcr-egfp* cassette introduced by pCM80-mcr-egfp	This study
AM1-M80	Transformant of *M. extorquens* AM1 with plasmid-dependent *mcr* introduced by pCM80-mcr	This study
AM1-MGTC	First-round *mcr* chromosomal integration recombinant generated by transforming pTP-cre-mcr-egfp into *M. extorquens* AM1, containing the *mcr-egfp* cassette and inducible *cre*.	This study
AM1-MGT	Single *mcr* chromosomal integration recombinant generated by transforming pTP-mcr-egfp into *M. extorquens* AM1, containing the *mcr-egfp* cassette without inducible *cre*.	This study
AM1-2MGTC	Second-round *mcr* chromosomal insertion recombinant obtained by re-transformation of pTP-cre-mcr-egfp into the AM1-MGTC after induction.	This study
AM1-DMG	Combination expression recombinant obtained by transforming pTP-cre-mcr-egfp into AM1-MG80	This study
AM1-2DMG	Combination expression recombinant obtained by transforming pCM80-mcr-egfp into AM1-2MGTC	This study

### Construction of the integration vector pSL-TP-cre-km with polynary elements of *cre*-*lox71/66* and *miniHimar1* transposon

The *cre* gene, which was amplified from pCM157 using primer pairs Cre-F (5’-ggaAGATCTATGTCCAATTTACTGACCGTAC-3’) and Cre-R (5’-ccgGAATTCCTAATCGCCATCTTCCAGC-3’) (restriction sites *Bgl*II and *EcoR*I are underlined), was cloned into pLC291 to form pLC-Cre. The *miniHimar1* transposon elements (*IR-TP-IR*), which was amplified from p15A-M6.8 using primer pairs TP-F (5’-cgcGGATCCCGCCTTCTTGACGAGTTC-3’) (restriction site sequence for BamHI are underlined) and TP-R (5’- ATTCCGGAGTATACGTAGCC − 3’), was cloned into pSL1180 to create pSL-TP.

The *lox71* and *lox66* were incorporated into the ends of forward and reverse primers, respectively: Lox71-pro-F (5’-ccGTTAAC*TACCGTTCGTATAGCATACATTATACGAAGTTAT*CTGGCACGACAGGTTTCC-3’) and Lox66-km-R (5’-ggACTAGT*TACCGTTCGTATAATGTATGCTATACGAAGTTAT*CATAAACAGTAATACAAGGGGTG-3’) (restriction sites HpaI and SpeI are underlined and the *lox71* and *lox66* sites are italicized). Then the 4.2-kb fragment, comprising a terminator, a *PR/tetO* inducible promoter, a *cre* gene and a kanamycin antibiotic gene, was amplified from pLC-Cre using primer pairs Lox71-pro-F and Lox66-km-R. The resulting fragment was cloned into pSL-TP to create the polynary plasmid pSL-TP-cre-km (Fig. S2), which was used as the basic integrated vector for introducing the target gene later. Meanwhile, the 1.0-kb kanamycin gene fragment amplified from pLC291 using primer pairs of Km-F (5’-ccgGAATTCCGTCCCGTCAAGTCAGCGT-3’) and Km-R (5’-cccAAGCTTGCCATCCATCCCCGTGTC-3’) (restriction sites are underlined) was cloned into the corresponding region of pSL1180 to form pSL-km, as a control plasmid.

### Construction of vectors with co-transcribed *mcr* and fluorescent report gene *egfp*

In this study, the *mcr* gene, which was amplified using primer pairs mcr-F (5’-cccAAGCTTCTCGAGATGAGCGGAACAGGACGAC-3’) and mcr-R (5’-cgcGGATCCCTCGAGTTACACGGTAATCGCCCGTC-3’), was transferred into pCM80 to generate pCM80-mcr. The promoterless fluorescent gene *egfp* was designed to locate afterward *mcr* forming a co-transcript. The corresponding plasmid pCM80-mcr-egfp was constructed by ligating the 7.6-kb *Nhe*I-*BamH*I fragment from pCM80-eGFP with the 4.0-kb *Nhe*I-*BamH*I fragment from pCM80-mcr (Fig. S3).

For further construction, the homo-tail enzymes *Spe*I and *Nhe*I were used to combine all the necessary elements. Specifically, the *Spe*I digested and lined fragment of pSL-TP-cre-km, was combined with the *Nhe*I-digested fragment of pCM80-mcr-egfp. The resulting vector was named pCM-TP-cre-mcr-egfp and then digested with *Sfi*I to remove *oriV* replicon origin. The remaining 17-kb fragment was self-ligated to generate the target multiple insertion plasmid, which was named pTP-cre-mcr-egfp. Meanwhile, the 4.7 kb *Nhe*I-*EcoR*I fragment from pCM80-mcr-egfp was blunted and then ligated into *Sma*I-lined fragment of p15A-M6.8 to create a single insertion plasmid, which was named pTP-mcr-egfp and served as a control (Fig. S4).

### Electroporation of *M. extorquens* AM1

The plasmids were transformed into *M. extorquens* AM1 by electroporation as described in a previous study [53], with a few adjustments. Initially, *M. extorquens* AM1 cells were grown in a 50-mL flask until the culture reached OD_600_ of 0.8. The cells were then collected, washed thrice with ice-cold water to prepare competent cells, and resuspended in 1 mL ddH_2_O for distribution. Next, 100 µL of competent cells mixed with 2 µg of plasmid DNA were transferred into a 1 mm-gap cuvette placed on ice and operated at a voltage of 1800 V using the electroporator (Eppendorf, Germany). The mixture was transferred into 2 mL liquid medium in a 10 mL tube and incubated at 30℃ with a rotational speed of 200 rpm for 5 h. 100 µL of the culture was subsequently spread onto selective plates. The objective colonies appeared after 3–5 days via antibiotic resistance, which were confirmed by colony PCR.

### Confirmation of the *miniHimar1* transposon function

The transposon-containing plasmids pSL-TP-cre-km and p15A-M6.8 were individually transferred into *M. extorquens* AM1 to detect the transformants in the kanamycin-selection plate. In contrast, the transposon-free plasmid pSL-km was used as a negative control. The resultant transformants obtained from the transformation of p15A-M6.8 were identified as *M. extorquens* AM1(*lacZ*). The plasmid pSL-TP-cre-km was then transformed into *M. extorquens* AM1(*lacZ*) to detect transposon immunity.

### Functional assay of the *cre*-*lox71/66* system and the second round of chromosomal integration

The plasmid pSL-TP-cre-km was transformed into *M. extorquens* AM1 to investigate the function of the *cre*-*lox71/66* system., The obtained transformants were initially incubated in a km-free liquid medium till OD_600_ reached 0.8. Subsequently, aTc was added at a final concentration of 25 ng/mL for 2–3days to induce Cre expression, facilitating the removal of the regions between *lox71* and *lox66* sites, which contained the kanamycin resistance gene. After induction, an aliquot (50 µL) was spread onto a plate and incubated at 30 °C. Single colonies were picked and screened for kanamycin resistance. The recombination efficiency of Cre was calculated by dividing the number of total colonies by the number of kanamycin-sensitive colonies present.

Following the first round of electroporation, the transformants were examined for detecting the gene expression levels, and the isolate that exhibited the desired traits was selected as the host of the second round. Then, the host was induced by aTc to initiate recombination by *cre*-lox71/lox66 elements, and the resulting kanamycin-sensitive clones were screened to ensure successful recombination. This process was repeated until the anticipated colonies with a second copy of the target genes were obtained.

### Detection of relative green fluorescence intensity

The selected *M. extorquens* colonies with *egfp* expression were cultured for 2 days until OD_600_ reached approximately 0.8 in the tube with the medium at 30℃. Subsequently, the cultures were diluted into 50 mL of the medium at an initial OD_600_ of 0.01 in shaking flasks. The optical density and fluorescence were measured at designated intervals over 60 h period. The expression levels of green fluorescent protein were quantified using a BioTek Cytation I cell imaging microaperture plate detection system (BioTek, USA). For each measurement, 200 µL of each strain culture was added into each well of the 96-well plate for fluorescence reader (GFP: excitation 485/20 nm, emission 528/20 nm) and absorbance at 600 nm (OD_600_). The absorbance at 900 and 977 nm (OD_900_ andOD_977_) was recorded once to correct the optical path [54]. The negative control strain was included in every plate and its fluorescence was subtracted for normalization. The fluorescence intensity (also referred to as fluorescein activity) was calculated as fluorescence/OD_600_ at each time point [55]. Each culture was performed in triplicates.

### 3-Hydroxypropionic acid quantification

The quantification of 3-HP was performed using the method described in a previous method [4]. Briefly, 100 mL of cell culture with an OD_600_ between 1.0 ± 0.1 was collected by centrifuging at 10,000×*g* for 10 min. The supernatant was used as a sample for 3-HP detection. The concentration of 3-HP was determined by LC–MS carried out using an Agilent LCQQQ-MS system (Agilent 1290 Infinity-6460, Agilent Corp. CA, USA) in the negative-ion mode. An aliquot of 10 µL of the sample was injected and separated on an Agilent SB C18 column (100 × 2.1 mm, 1.8 μm) at a temperature of 35 °C, with mobile phase A consisting of 10% (*v*/*v)* buffer in water and mobile phase B consisting 10% (*v*/*v*) buffer in acetonitrile. The buffer consisted of 200 mM formic acid adjusted to pH4.0 with ammonium hydroxide solution. The linear gradient was as follows: 0–3 min, 5–10% B; 3–5 min, 100% B; 5–7 min, 5% B at a flow rate of 0.2 mL/min. 3-HP was identified by comparison to the retention time, 249 nm of ultraviolet (UV) spectra, and the MS2 pattern of the authentic reference standard sample, which was purchased from the Tokyo Chemical Industry (Tokyo, Japan). Each titer was measured in biological triplicates.

### RNA extraction and qRT-PCR analysis

Total RNA of *M. extorquens* strain samples were extracted from 3 mL of cells in the mid-exponential phase (OD_600_ of 1.0 ± 0.1) using the TransZol Up RNAspin Mini Kit (TransGen Biotech, Beijing, China) following the manufacturer’s instruction. The RNA concentration was measured using a NanoDrop One Microvolume UV-Vis Spectrophotometer (Thermo Fisher Scientific, MA, USA). The cDNA library was prepared using a HiScrip IIIQRT SuperMix kit for qPCR (Vazyme Biotech Co., Ltd., Nanjing, China) following the manufacturer’s instruction. The reaction system was prepared using AceQ Universal SYBR qPCR Master Mix (Vazyme, Nanjing, China) following the manufacturer’s protocol in a total volume of 10 µL for each tube. Further, qPCR was performed using QuantStudio 5 ABI 7500 real-time PCR System (Thermo Fisher Scientific). Genes encoding 16S ribosomal protein (*rpsB*) were used as internal control genes. The relative expression levels were calculated using the relative 2^−(ΔΔCt)^ method, and the expression level was normalized to the expression level of the control genes. The primer pairs used for qRT-PCR were as follows: *rpsB*-F (5’-CACCCGCAACAACATCCAC-3’) and *rpsB*-R (5’-GTCTTCCAGTTGGTCAGCATG-3’) used for *rpsB*; and *mcr-*F (5’-ACAGGACGACTGGCAGGAAAG-3’) and *mcr-*R (5’-GAATCTCGGCCAGACGACGC-3’) used for *mcr*.

### Statistical analysis

Standard curves, GFP expression, and 3-HP production were analyzed using GraphPad Prism 8.0 software and Excel 2016 in this study. Statistical analysis was performed using the Student *t* test. Data were presented as the mean ± SDs (standard deviation); ** *p* < 0.01.

### Electronic supplementary material

Below is the link to the electronic supplementary material.

Supplementary Material 1

## Data Availability

All data generated and analyzed in the present study are included in this manuscript and its additional files, and are also available from the corresponding author upon reasonable request.
