# Supplementary material for: Heterologous production of 3-hydroxypropionic acid in Methylorubrum extorquens by introducing the mcr gene via a multi-round chromosomal integration system based on cre-lox71/lox66 and transposon
Source: Microb Cell Fact. 2024 Jan 3;23:5. doi: 10.1186/s12934-023-02275-z (PMC10763676; doi:10.1186/s12934-023-02275-z)

**Supplementary data**

**Heterologous production of** **3-hydroxypropionic acid in *Methylorubrum extorquens* by introducing the *mcr* gene via** **a** **multi-round chromosomal integration system based on *cre-lox71*/*lox66* and transposon**

Li-ping Zhu, Ya-zhen Song, Shu-nan Ma, Song Yang

**Fig. S1 Map of vectors constructed in this study.**

**(a)** pSL-TP-cre-km; **(b)** pCM80-mcr-egfp; **(c)** pTP-cre-mcr-egfp; **(d)** pTp-mcr-egfp.

**Fig. S2 Construction of plasmid pSL-TP-cre-km.**

**(a)** Schematic construction strategy of plasmid pSL-TP-cre-km and the restriction enzymes used for digestion and ligation in each step are marked in red. **(b)** Gel electrophoresis analysis of the plasmids generated in each step. M1 stands for DL15000 DNA marker and M2 for supercolid marker. 1 stands for the PCR fragment of *cre* from pCM157 with its 5’ and 3’ends digested by *Bgl*Ⅱ and *EcoR*Ⅰ. 2 stands for liner pLC291 digested by *Bgl*Ⅱ and *EcoR*Ⅰ. 3 stands for the plasmid pLC-Cre constructed by ligation of 1 and 2. 4 stands for the PCR fragment of *Tpase* from p15A-M6.8 with its 5’ and 3’ends digested by *BamH*Ⅰ and *Xba*Ⅰ. 5 stands for liner pSL1180 digested by *BamH*Ⅰ and *Xba*Ⅰ. 6 stands for the plasmid pSL-TP constructed by ligation of 4 and 5. 7 stands for the PCR fragment of *Cre-km-lox71/lox66* from pLC-Cre with its 5’ and 3’ends digested by *Hpa*Ⅰ and *Spe*Ⅰ. 8 stands for the liner pSL-TP digested by *SnaB*Ⅰ and *Spe*Ⅰ. 9 stands for the plasmid pSL-TP-cre-km constructed by ligation of 7 and 8.

**Fig. S3 Schematic construction strategy used to generate plasmid pCM80-mcr-egfp.** The restriction enzymes used for digestion and ligation are marked in red.

**Fig. S4 Schematic construction of plasmid pTP-cre-mcr-egfp and pTP-mcr-egfp and the gel electrophoresis analysis of the plasmid pTp-cre-mcr-egfp.** The restriction enzymes used for digestion and ligation in each step are marked in red.

M1 stands for DL15000 DNA marker and M2 for DL5000 DNA marker. The plasmid pTP-cre-mcr-egfp and its digestion with *EcoR*Ⅰ are showed as 1 and 2, respectively.

**Fig. S5 Detection of inducible *cre-lox66/lox71* system in *E. coli* and *M. extorquens* AM1. (a)** Plasmid extraction and *EcoR*Ⅰ digestion assay after induction in pSL-TP-cre-km-harboring *E. coli* host. Ⅰ indicates the length (9.1kb) of original pSL-TP-cre-km as shown with an asterisk. Ⅱ indicates the length (5.2kb) of the smaller plasmid derived by recombination as expected. Then *EcoR*Ⅰ digestion assay (on the right) for the smaller recombinant plasmid. The smaller plasmid was digested by *EcoR*Ⅰ to be fragments of 3.5k, 1.2k and 0.5k bp as shown in lane 1 and 2, while the pSL-TP-cre-km to be fragments of 4.5kb, 2.9kb, 1.2kb and 0.5kb as shown in lane 3, respectively. M1, M2 and M3 refer supercoiled DNA marker, DL15000 DNA marker and DL5000 DNA marker, respectively. **(b)** After induction of culture of *M. extorquens* AM1-TC, the colonies present on the non-kanamycin plate were picked for inoculating on the plates with (km+) and without (km-) kanamycin correspondingly. Total colonies were counted and the efficiency of recombination was calculated accordingly. **(c)** colony-PCR amplification verification of excision of the gene fragments between *lox71* and *lox66*. The primer pairs used for each section: Ⅰ with Lox71-pro-F / Lox66-km-R; Ⅱ with Cre-F/km-test-R; Ⅲ withTP-test-F/Cre-R. The templates used for each sample were as follows: 1 indicates pSL-TP-cre-km plasmid, 2 indicates the strain *M. extorquens* AM1-TC. 3-6 indicate the colonies of *M. extorquens* AM1-T. M3: DL5000 DNA marker, M4: DL5000 plus DNA marker. The red arrows indicate the positive fragments with length of 4.2kb, 1.5kb, 3.2kb, respectively. **(d)** The growth curve of the strains of *M. extorquens* AM1, *M. extorquens* AM1-TC and *M. extorquens* AM1-T.

**Fig. S6** HPLC analyses of 3-HP production ability of the strain AM1-MG80. Row 1, as a negative control, shows the analysis for the wild type *M. extorquens* AM1without the *mcr* gene; row 2, as a positive control, shows that of the standard 3-HP with a peck appeared at 13.5 min; the rows 3 and 4 show the same analysis for the isolates of AM1-MG80 and AM1-M80. The horizontal axis Min represents time of minute and the vertical axis AU represents the absorbance unit. The red arrow indicates the peak of 3-HP.

**Fig. S7 Effect of aTc concentration on induction efficiency of the *cre-lox71/lox66* in the recombinant AM1-MGTC.**

**(a)** Induction efficiency of the *cre-lox71/lox66* under the 25 ng/mL and 50 ng/mL of aTc, respectively. **(b)** The location of TetR binding sites in the chromosome of AM1-MGTC. TerR was encoded by *tetR* gene as the red arrows indicate.

Fig. S1


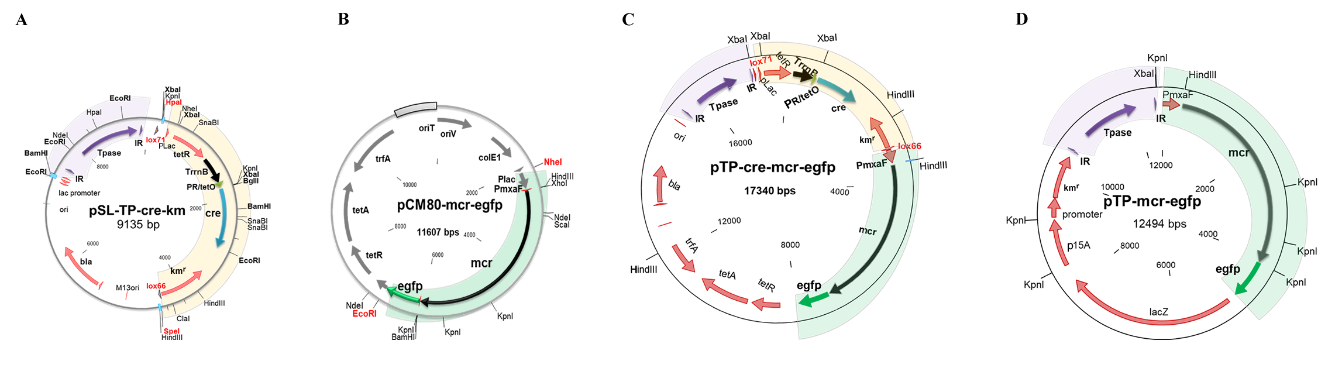


Fig. S2





Fig. S3





Fig. S4


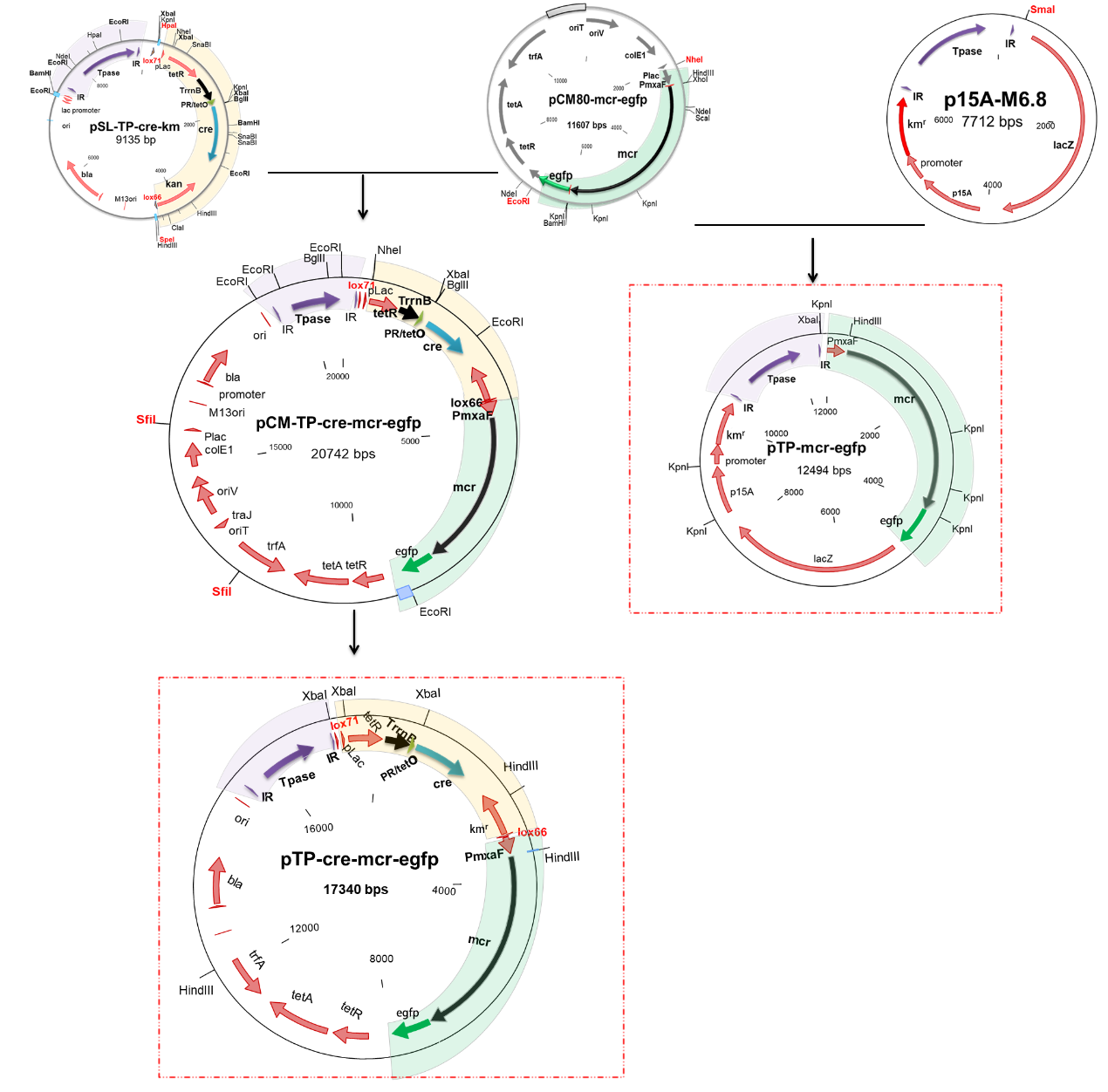


Fig. S5


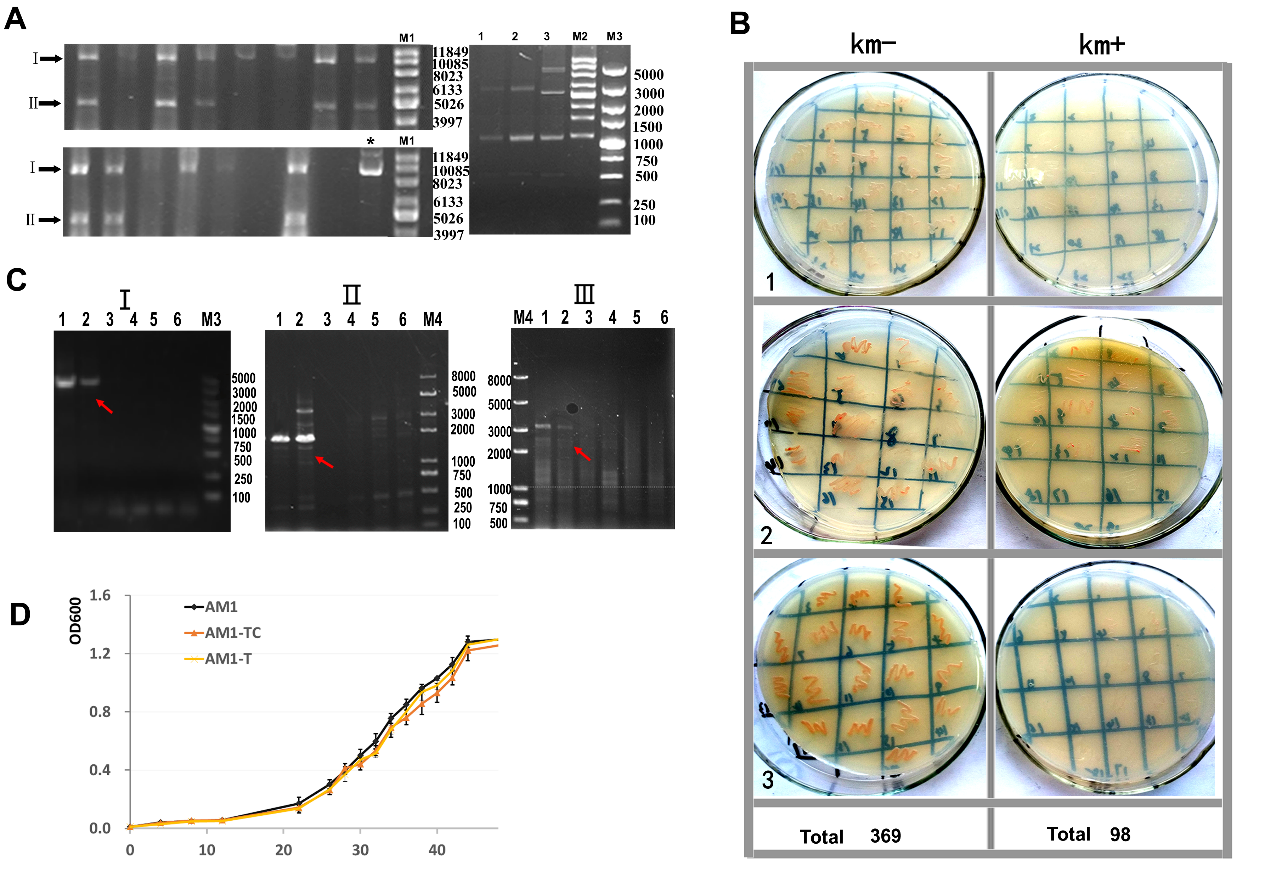


Fig. S6


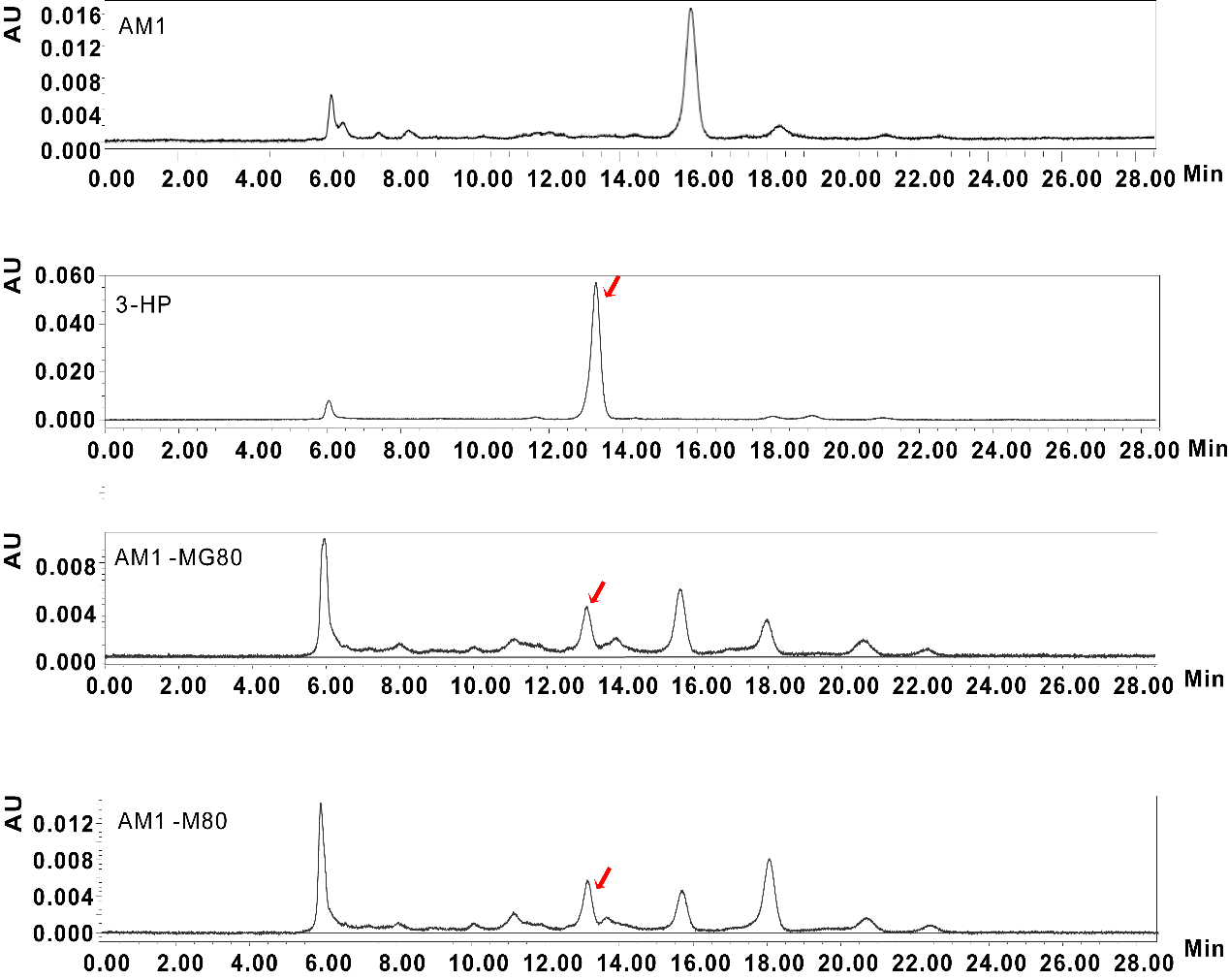


Fig. S7


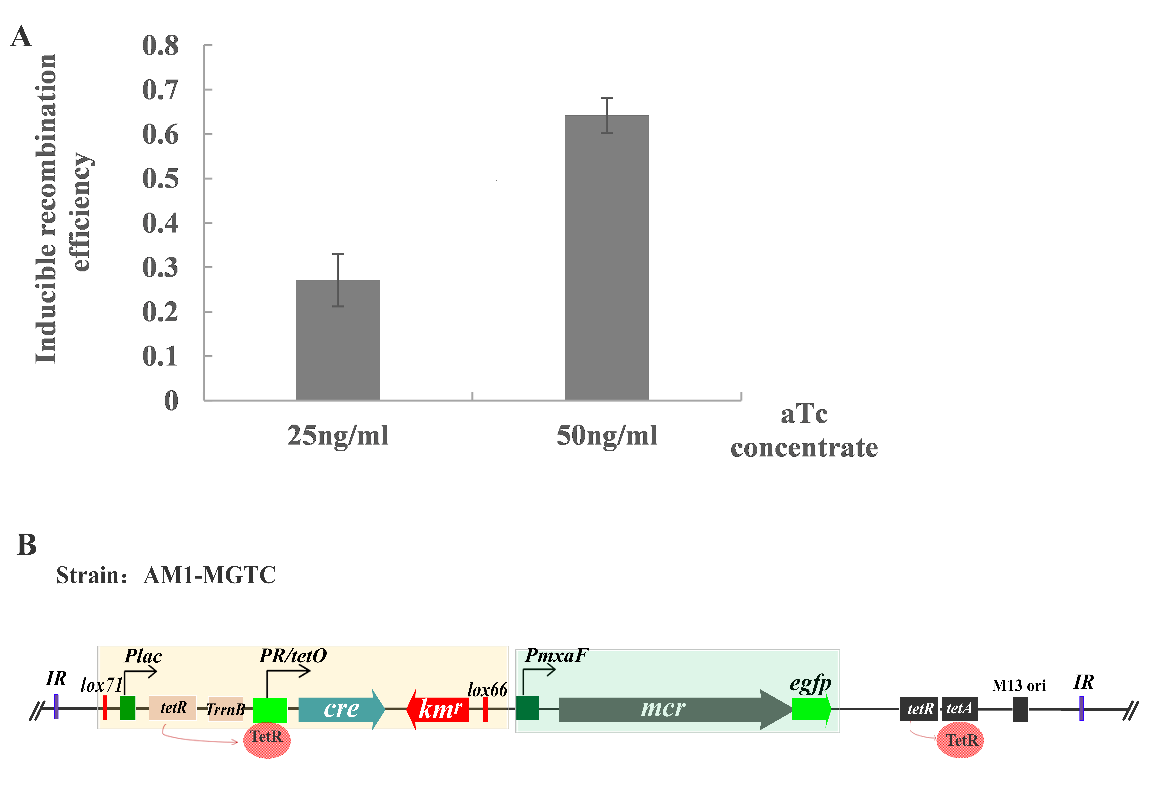

Supplement: Supplementary file 1 — Supplementary Material 1 [file 12934_2023_2275_MOESM1_ESM.docx]
